# Immunotherapies and Targeted Therapies in the Treatment of Metastatic Colorectal Cancer

**DOI:** 10.3390/medsci7080083

**Published:** 2019-07-30

**Authors:** Prashanth Rawla, Adam Barsouk, Andreas V. Hadjinicolaou, Alexander Barsouk

**Affiliations:** 1 Department of Medicine, Sovah Health, Martinsville, VA 24112, USA; 2 Hillman Cancer Center, University of Pittsburgh, PA 15232, USA; 3 Academic Clinical Post-doctoral Fellow and Gastroenterology Resident, MRC Cancer Unit and Department of Gastroenterology, University of Cambridge, Cambridge CB2 0XZ, UK; 4 Hematologist-Oncologist, Allegheny Health Network, Pittsburgh, PA 15212, USA

**Keywords:** colorectal, metastatic, targeted therapy, epidermal growth factor receptor inhibitors, novel therapeutics

## Abstract

Colorectal cancer (CRC) is the third leading cause of cancer deaths, and while mortality has largely improved in the developed world, five-year survival for metastatic disease remains dismally low at only 15%. Fortunately, nearly a dozen targeted therapies and immunotherapies have been FDA approved in the past decade for certain patient profiles with metastatic CRC (mCRC), and many others are under development. Checkpoint inhibitors such as pembrolizumab have proven effective at extending survival for mismatch repair (MMR)-deficient and high microsatellite instability (MSI) mCRC patients. In combination with chemotherapy in first- and second-line treatment, antiangiogenic (anti-vascular endothelial growth factor (anti-VGEF)) agent bevacizumab has been shown to increase mCRC survival. Anti-epidermal growth factor receptor (anti-EGFR) agents panitumumab and cetuximab, in combination with chemotherapy, have also prolonged survival among *KRAS* and all *RAS* wild-type mCRC patients. Among these patients, anti-EGFR therapy has been found to be more efficacious than bevacizumab. Improved selectivity has allowed small-molecule receptor tyrosine kinase (RTK) inhibitors to target VEGF and EGFR with greater efficacy and tolerability. Combinations of immunotherapies, RTKs, monoclonal antibodies, and cytotoxic drugs are being investigated to provide broad-spectrum protection against relapse by simultaneously targeting many cancer hallmarks. Lastly, human epidermal growth factor receptor 2 (HER2) therapy has shown promise for HER2-positive mCRC patients, though larger clinical trials are required to secure FDA approval.

## 1. Introduction

With nearly two million new cases and one million fatalities in 2018, colorectal cancer (CRC) is the third leading cause of cancer deaths around the world [1,2]. Moreover, the global burden of the disease is expected to increase by a further 60% by the year 2030 [3]. Much of the growth in incidence is associated with the economic development of transitioning nations, which often goes hand in hand with a transition to the “Western” diet and lifestyle. Obesity, physical inactivity, and consumption of red and processed meat, alcohol, and tobacco have all been identified as significant risk factors for CRC [3]. In addition to the environmental risk factors, genetic and epigenetic aberrations are very prominent in colorectal cancer leading to initiation, propagation, and dissemination of the tumor [4]. Environmental, epigenetic, and genetic risk factors can in fact often crosstalk in the context of carcinogenesis. The significant genomic instability generated by such modifications means that identifying specific genetic and epigenetic alterations can allow a targeted and precise approach to therapeutic targets disclosure [4].

Beyond the genes altered sequentially as part of the of the adenoma–carcinoma sequence such as the oncogenes, *APC* and *KRAS*, and tumor suppressor genes, *TP53* and deleted in colorectal carcinoma (*DCC*), a significant proportion of colorectal cancers display high degrees of microsatellite instability (MSI) as a result of defects in genes involved in the DNA mismatch repair (MMR) pathway such as *MLH1, MSH2, MSH6,* and *PMS2* [5]. Tumors with high MSI are more prone to accumulating mutations that can often generate neoantigens recognized by the immune systems. This principle is crucial for the purposes of this review as the majority of CRCs responding to immunotherapy have a high mutation rate. The main immunotherapy targets are programmable death protein 1 (PD-1) and cytotoxic T-lymphocyte-associated protein 4 (CTLA-4), checkpoint molecules that regulate the function of tumor-specific T-cells. On the other hand, targeted therapies aim mainly at tyrosine kinases such as vascular endothelial growth factor (VEGF), epidermal growth factor receptor (EGFR), placental growth factor (PGF), and human epidermal growth factor receptor 2 (HER2). These therapies block growth factor signaling pathways that would otherwise aberrantly instruct tumor cells to continuously grow, divide, and metastasize via angiogenesis. As such, the effect of monoclonal antibodies against these kinases often depends on the *KRAS* or *BRAF* status of the tumor, which is another reason why understanding the molecular pathogenesis of the cancer is crucial in deciding the appropriate treatment [5].

Even in the face of this growing incidence, CRC mortality in developed nations such as the US and Western Europe has fallen thanks to improvements in early diagnosis and treatment [3,6]. Procedures available for screening and early diagnosis include colonoscopy, flexible sigmoidoscopy, computed tomography (CT) colonography, fecal immunochemistry, and fecal occult blood testing [7]. Cases diagnosed in Stage I have a 92% five-year survival, as compared to a 15% five-year survival for metastatic (stage IV) cases in the US [6]. Early diagnosis in developed countries may also contribute to the increasing incidence rates [3].

Nevertheless, survival rates have also improved among those with advanced stage disease [6], indicating advances not only in early diagnosis but also in the treatment of metastatic CRC (mCRC) itself. While surgery remains the primary option for localized disease, a wide array of therapeutics has been developed to slow the progression of late-stage CRC. Multi-drug chemotherapeutic regimens such as FOLFIRI (folinic acid, fluorouracil and irinotecan), FOLFOX (folinic acid, fluorouracil and oxaliplatin), and CapeOX (capecitabine plus oxaliplatin) have become the standard of care, disrupting multiple metabolic and proliferative pathways to avoid or delay cancer resistance or relapse [8]. Identification of genetic mutations that drive CRC tumorigenesis has enabled the development of targeted therapies and immunotherapies that, in certain patient profiles, have displayed better efficacy for mCRC, and fewer adverse events, compared to traditional chemotherapy regimens. Immunotherapies and targeted therapies are currently being investigated in broad-spectrum combinations, including partnerships with established chemotherapeutics in an effort to address multiple hallmarks of cancer and, thus, lower the risk of recurrence and relapse [9]. Another avenue of recent development lies in the field of “migrastatics”—drugs that target cancer invasion and metastasis, which account for over 90% of cancer mortalities [10]. This is a phenomenon observed in most cancers whereby tumor cells evolve; the adaptive mechanisms that allow them to employ their cytoskeleton in order to invade the extracellular matrix and metastasize. Technologies such as small-molecule inhibitors targeting receptor tyrosine kinases (RTKs) can be employed against molecules involved in invasion and metastasis when overexpressed by cancers [10].

## 2. Immunotherapy

Immunotherapy involves priming the host’s natural immune defenses to recognize, target, and destroy cancer cells effectively. Like other tumors, CRC has been shown to evade immune detection through several mechanisms. Firstly, CRC inhibits circulating cytotoxic T-cells by releasing immunosuppressive cytokines such as Transforming growth factor beta, as well as by recruiting immunosuppressive cell populations, such as regulatory T-cells and myeloid-derived suppressor cells (MDSCs) to the tumor microenvironment [11,12]. Additionally, CRC tumorigenesis appears to be sustained by inflammation, as seen in conditions such as inflammatory bowel disease, and CRC further releases inflammatory cytokines such as IL-6 to increase blood flow and tumor growth and evade immunity [13,14]. Other methods of immunosuppression include antigen presentation, trafficking, and recognition of tumor cells via major histocompatibility complex downregulation. 

Immunotherapies include vaccines, checkpoint-inhibitors, and other agents that have shown unprecedented efficacy in the treatment of other advanced cancers. However, not all immunotherapies have proven equally effective against CRC. Levamisole, an immunomodulating agent which helps promote antibody production against tumor antigens, did not demonstrate efficacy as an adjuvant CRC monotherapy [15,16]. However, the combination of levamisole and 5-FU displayed equal efficacy with the current standard of 5-FU and leucovorin [17]. Several similar agents have been under development but have not proceeded to later-phase clinical trials. 

## 3. Checkpoint Inhibitors

Checkpoint inhibitors are a class of immunotherapies that function by targeting and blocking cancer’s attempt to suppress T-cell detection [18]. Cancer cells exploit weaknesses in the natural regulation of T-cells by inducing the expression of co-inhibitory signals such as cytotoxic T-lymphocyte-associated protein 4 (CTLA-4) or programmed death ligand 1 (PD-L1) on tumor-infiltrating lymphocytes. These receptors normally act as natural checkpoints to control the activity of T-cells, for example, during the clearance of a viral infection, in order to prevent damage to the host. However, in a cancer context, engagement of these T-cell receptors by ligands expressed on cancer cells propagates an inhibitory signal that induces the circulating cytotoxic T-cells to ignore tumor cells and become anergic, or even commit apoptosis, thus allowing CRC to avoid the body’s immune defenses [19]. In turn, checkpoint inhibitors function to block these receptors and thus the mitigation of the negative regulatory signals (during T-cell priming and exposure to tumor antigen), enabling the unrestricted activity of tumor-specific T-cells, and have proven remarkably effective in patients with certain mCRC sub-types. 

Specifically, mismatch repair (MMR)-deficient cancers, which comprise about 15% of all CRCs, are highly responsive to immune checkpoint blockade therapy [20,21]. By promoting frameshift mutations, MMR deficiency results in cancer cells displaying unusual antigens, known as neoantigens, on their surface. Since host T-cells have acquired no immunological tolerance for these neoantigens, MMR-deficient CRC is more likely to be recognized by the immune system, labeled as foreign/dangerous and targeted for destruction. As these rapidly mutating cancers are also more likely to express ligands (such as PD-L1) to engage the immunosuppressive checkpoint molecules on T-cells, they are excellent targets for treatment with checkpoint inhibitors [22]. For all these reasons, MSI, a characteristic feature of CRC, increases the efficacy of checkpoint inhibitors [23]. 

In fact, in 2017, pembrolizumab (Keytruda), became the first immunotherapy to be approved by the FDA for metastatic or unresectable CRC, specifically tumors with high MSI or poor MMR. Keytruda is a monoclonal antibody against PD-1 (programmed death protein 1) that acts as a checkpoint inhibitor by binding PD-1 on cytotoxic T-cells and preventing their interaction with the immunosuppressive PD-L1 and PD-L2 ligands overexpressed in cancer cells [24,25]. A phase II trial found Keytruda to be more effective than the standard of care for MMR-deficient CRC. The trial reported an objective response rate (ORR) and progression-free survival (PFS) of 40% and 78%, respectively, for patients with MMR-deficient CRC, as compared with 0% and 11% seen in patients with MMR-proficient CRC [26]. 

The other two FDA-approved immunotherapy options for CRC are PD-1 inhibitor nivolumab (i.e., Opdivo) monotherapy or a combination of nivolumab and ipilimumab (Yervoy) [27]. Ipilimumab is a humanized monoclonal antibody that binds CTLA-4, a T-cell receptor that also inhibits immune stimulation. Blocking CTLA-4 removes the inhibitory signal received by T-cells during their antigen-specific stimulation and allows them to activate and target cancer cells for destruction. While an initial phase I trial found nivolumab ineffective for CRC, a complete response was observed in one patient with high MSI and PD-L1 expression [28]. In a later phase II study (CheckMate 142), nivolumab proved effective for those who had progressed on three or more lines of therapy, with 68.9% of patients maintaining disease control for more than 12 weeks [29]. A subsequent phase II study found that the combination of nivolumab and ipilimumab for patients with deficient MisMatch Repair/ High levels of Micro Satellite Instability (dMMR/MSI-H) mCRC who had progressed on standard chemotherapeutic regimens had an ORR of 55% (median follow-up of 13.4 months), with disease control for 12 weeks or more in 80% of the cohort and 12-month PFS of 71% and 12-month overall survival of 85% [30,31]. Unsurprisingly, checkpoint inhibitors have been repeatedly shown to be ineffective in microsatellite-stable (MSS) and refractory canonical CRC [28,30,32,33].

Other checkpoint inhibitors currently under investigation target molecules such as Lymphocyte activation gene-3 *(LAG-3*) and T cell immunoglobulin-3 (*TIM-3*). Broad spectrum combinations of checkpoint inhibitors, along with proven chemotherapeutics, are being investigated for MMR-proficient patients, the more common subset of mCRC, and the one that has not yet benefited from checkpoint inhibition [34]. Studies are being conducted on the viability of using nanoparticle-based chemotherapy to sensitize tumors to immune checkpoint blockade and promote antitumor immunity [35].

## 4. Other Immunotherapies

Cancer “vaccination” involves injecting the patient with select tumor antigens to stimulate immune-mediated recognition of cancers. Autologous vaccination, which involves infusing the patient with their own identified tumor antigens, has not proven effective for CRC in several clinical trials [36,37]. Similarly, neither peptide-based vaccines, which present a unique and specific portion of the cancer cell antigen to the immune system, nor dendritic cell vaccines [38,39,40], which aim to prime the body’s primary antigen presenting cells (APCs) with cancer antigens, have shown efficacy as a CRC treatment [41,42]. 

Adoptive cell transfer, another type of immunotherapy, aims to boost the immune response against cancer by replenishing the host’s own (often cancer-specific) T-cell levels through ex vivo expansion and re-injection in the patient. A phase II trial for adjuvant therapy found that expanded sentinel lymph node (SLN)-T lymphocytes could boost OS from 14 to 28 months, prompting further investigation [43]. Toll-like receptors (TLR) promote cancer recognition through activation of the innate immune system, and while TLR9 agonists were found effective in mouse models, a mCRC maintenance trial found only modest improvement in human patients [44]. Nevertheless, these immunomodulating agents are being investigated in combination with other immunotherapies, targeted therapies, and chemotherapies. Novel therapies currently under investigation include inhibitors against enzymes that generate nutritional stress for T-cells such as indoleamine 2,3-dioxygenase (IDO) and immunomodulatory molecules such as CD73 and A2A receptors [45,46]. Additionally, early phase studies looking at a novel T-cell bispecific antibody targeting both Carcinoembryonic antigen (CEA) on tumor cells and CD3 on T-cells had shown evidence of anti-tumor response in mCRC patients following monotherapy regimes, and further response enhancement when anti-CEA/CD3 was combined with the anti-PD-L1 checkpoint inhibitor atezolizumab [47]. 

Finally, the microbiome has been shown to play an important role in modulating the immune response to CRC, and the effects of chemotherapy and immunotherapy on the microbiome are currently under investigation in clinical trials [48,49].

## 5. Targeted Therapies

### 5.1. Anti-Angiogenic Agents

Angiogenesis refers to the formation of a new vascular network by endothelial cell budding and growth of pre-existing vasculature. While angiogenesis is an essential process to keep cells oxygenated, it is often abused by cancer cells to increase access to nutrients and also to invade the bloodstream and metastasize to distant sites [50]. Anti-angiogenic agents can be used to inhibit cancer growth and metastasis, as well as to improve circulation of other cytotoxic agents to all parts of cancer by “normalizing” the surrounding vasculature [51]. 

Vascular endothelial growth factor (VEGF) is the primary cytokine that has been implicated in tumor-induced angiogenesis. The VEGF family of proteins is composed of five receptor tyrosine kinases (RTK), of which VEGF receptor-2 (VEGFR-2) is the main member involved in the formation of new blood vessels [52]. Bevacizumab is a monoclonal antibody targeting VEGFR-2 [53]. It was first approved by the FDA in 2004 for mCRC in combination with other cytotoxic agents. By blocking VEGFR-2, bevacizumab prevents cancer angiogenesis.

In its first clinical trial, the addition of bevacizumab to first-line treatment with 5-FU chemotherapy significantly improved overall survival (OS) (20.3 vs. 15.6 months), PFS (10.6 vs. 6.2 months), and RR (44.8% vs. 34.8%) [54]. A later phase III trial found that bevacizumab plus FOLFIRI chemotherapy also produced significant improvement in PFS and OS [55]. Another trial investigating the combination of bevacizumab with oxaliplatin (platinum-based) chemotherapy found significant prolongation only in PFS when compared to just oxaliplatin [56]. A recent trial evaluated a combination of all cytotoxic agents known as FOLFOXIRI with and without bevacizumab. While the addition of bevacizumab improved PFS and RR, it also increased the incidence of grade 3/4 (significant) adverse events [57]. As an anti-angiogenic agent, bevacizumab has been associated with vascular-related adverse events such as hypertension, bleeding, and thromboembolic events. It is contraindicated for patients with high blood pressure and coronary artery disease [58]. A study of safety in elderly patients found bevacizumab combined with capecitabine to be a well-tolerated regime [59]. Bevacizumab has also been shown to improve OS, PFS, and HR (hazard ratio of progression) as a second-line treatment and maintenance treatment for metastatic CRC [60,61]. 

Other FDA-approved anti-angiogenic agents for mCRC include aflibercept (which binds VEGF factor A and placental growth factor), ramucirumab (which targets VEGF and HER2/neu), and regorafenib [47,62]. The latter is an oral multi-RTK blocker that targets angiogenic pathways as well as oncogenic pathways and stromal suppressive molecules in the tumor microenvironment [63]. It demonstrated slightly prolonged OS and PFS compared to placebo in cases in which CRC had progressed after all approved therapies, but it also induced a higher incidence of adverse events, including fatal liver injury [64]. Apatinib, a small-molecule VEGF inhibitor that seems to prolong survival for gastric cancer is currently being investigated for mCRC [65]. Selective RTK inhibitors such as apatinib and regorafenib show improved selectivity as compared to early Tyrosine Kinase Inhibitors (TKIs) such as sorafenib, resulting in significantly better tolerability and improved safety profile. In order to overcome resistance, selective RTK inhibitors are being investigated in combination with immunotherapy and chemotherapy [66,67,68]. For example, in the context of chemotherapy-refractory or locally advanced mCRC, the combination of cobimetinib, a mitogen-activated protein kinase kinase (MEK) inhibitor with atezolizumab (an anti-PD-L1 checkpoint inhibitor) has so far displayed potentiation for anti-tumor T-cells to yield durable and improved OS, compared to those reported with standard of care regimes, with an acceptable safety profile [69].

### 5.2. EGFR Inhibitors

Epidermal growth factor receptor (EGFR) is a tyrosine kinase receptor involved in the signaling of cellular proliferation, angiogenesis, migration, and metastasis [70]. Predictably, it is upregulated in about 80% of colorectal cancers [71].

Cetuximab is a chimeric monoclonal antibody binding and inhibiting EGFR thus hindering cancer cell proliferation and growth as well as invasive potential and angiogenesis thus reducing the probability of metastasis. Cetuximab was originally approved for mCRC in 2004, though later experiments showed that the presence of *KRAS* mutation in cancer cells bestows resistance to cetuximab. As such, the therapy has since been confined to *KRAS* and *NRAS* wild-type (WT) mCRC patients [72]. Among WT mCRC patients in a large phase III trial, the addition of cetuximab to FOLFIRI significantly improved OS (23.5 vs. 20.0 months), PFS (9.9 vs. 8.4 months), and RR (57.3% vs. 39.7%) [73]. A phase II study of cetuximab in combination with FOLFOX-4 found improvements in RR and PFS but not in OS relative to FOLFOX-4 alone [74]. As similar results were not found in combinations of cetuximab with other chemotherapy regimens, the gold standard in first-line mCRC care has remained cetuximab with FOLFOX or FOLFIRI among *KRAS* WT cases [75,76].

In *KRAS* WT mCRC patients who had progressed under the standard of care, cetuximab used as second-line treatment was shown to significantly improve OS (9.5 vs. 4.8 months) and PFS (3.7 vs. 1.9 months) while preserving the quality of life standards as compared to best supportive care [77]. In the salvage setting, cetuximab plus irinotecan chemotherapy significantly improved PFS and RR, but not OS as compared to irinotecan monotherapy [78,79].

In similar fashion to cetuximab, panitumumab is a monoclonal antibody that targets EGFR and displays similar toxicities, namely skin rash and hypomagnesemia [80]. Although approved two years later, panitumumab has demonstrated similar efficacy to cetuximab when used in combination therapies [81,82]. However, panitumumab was not shown to be an effective salvage therapy for those who had progressed on cetuximab or had mutant *KRAS* [83,84,85].

### 5.3. Comparisons for Various Genetic Profiles

A phase II trial comparing targeted combination therapies found that for *KRAS* WT mCRC patients, panitumumab in combination with FOLFOX had similar PFS and RR but superior OS to that of bevacizumab with FOLFOX [86]. Similarly, a phase III trial found cetuximab plus FOLFIRI to have a superior OS compared to bevacizumab with FOLFIRI (of 28.7 vs. 25.0 months), with PFS and RR remaining the same between the two treatment options [87]. A separate study comparing the same treatment combinations found that cetuximab had superior RR, but not OS, against bevacizumab [88]. Likewise, panitumumab combination therapy was found to have better RR, but slightly lower PFS and OS, as compared to bevacizumab when used in second-line treatment [89]. 

While anti-EGFR therapy had long been considered the standard for KRAS WT CRC, a recent genomic analysis revealed that 17% of *KRAS* WT patients suffer from mutations in different *RAS* gene exons, including the *NRAS* gene [87]. Those cases that were WT for all these mutations (all-RAS) displayed prolonged OS with FOLFOX + anti-EGFR treatment as compared to those that were only *KRAS* WT [90]. In a retrospective analysis of the trials above, all-RAS WT patients were found to have further overall survival benefits (though questionable PFS benefits) under anti-EGFR combination therapy as compared to anti-VEGF (bevacizumab) [91]. Approved therapies and their associated biomarkers have been summarized in Table 1.

## 6. Targeted Therapies in Development

EGFR can also be blocked using small-molecule receptor tyrosine kinase (RTK) inhibitors such as gefitinib or erlotinib. Multiple phase II trials found that these RTK inhibitors, in combination with chemotherapy, produced no improvement in survival, but higher toxicities, as compared to standard chemotherapy [95,96,97]. Increasing the selectivity of RTK inhibitors has allowed for mitigation of adverse events and improved efficacy. Identification of new oncogenic targets beyond VEGF and EGFR for RTK inhibitors could further support this blossoming field and address the current limitations of targeted mCRC treatment [98]. Molecular profiling and identification of RTK activation patterns can allow for personalized, broad-spectrum multi-RTK therapies, which can be especially effective for single RTK-resistant mCRC cases [99].

For patients with mutant KRAS (thus unresponsive to anti-EGFR therapy), two other monoclonal antibodies for different surface receptors have been developed. Ganitumab targets type-1 insulin-like growth factor receptor, while conatumumab targets proapoptotic death receptor 5. In a trial in combination with FOLFIRI, conatumumab offered a slight improvement in PFS relative to ganitumab or placebo (6.5, 4.5, and 4.6 months, respectively), while the OS was unchanged [100]. 

A recent trial looking at trastuzumab (Herceptin) and lapatinib, two targeted therapies normally used for HER2-positive breast cancer, was conducted in the context of HER2-positive mCRC [101]. Trastuzumab was the first anti-cancer monoclonal antibody developed (for the HER2/neu receptor, a close relative of EGFR), while lapatinib is a small-molecule inhibitor of both the HER2/neu receptor and EGFR [102]. While only 5% of screened mCRC patients were identified as HER2-positive, the overall response rate (ORR) for those patients with this therapy was 30% [101]. Although promising for a small population, the therapy has yet to be tested in large clinical trials in order to be FDA approved.

## 7. Conclusions

As CRC incidence across the world continues to grow, development of better treatment options for the metastatic, late-stage disease has become even more imperative. CRC is the third leading cause of cancer mortality, and it continues to climb, while advanced-stage five-year survival has remained extremely low at only 15% in the US. Fortunately, several immunotherapies and targeted therapies have proven more efficacious and less toxic than the standard chemotherapy regimens for the treatment of mCRC.

Checkpoint inhibitors, a novel form of immunotherapy, have proven effective for MMR-deficient and high-MSI mCRC patients, while other forms of immunotherapy have yet to show significant promise. Anti-angiogenic agents such as bevacizumab have been shown to increase survival in first-line and second-line treatment in combination with chemotherapy, though they have a high rate of adverse events. Anti-EGFR agents improve survival among *KRAS* WT mCRC patients and offer an even greater benefit for all-RAS WT patients. For these patient groups, anti-EGFR therapy seems to be slightly more efficacious than bevacizumab. With improved selectivity, small-molecule RTKs may allow for safer and more efficacious targeting of *EGFR, VEGF*, and other mCRC oncogenes, such those promoting invasion and metastasis, that is yet to be fully characterized. While HER2 therapy was shown to improve survival among HER2-positive mCRC patients, larger clinical trials are required to investigate this benefit further, prior to approval.

## Figures and Tables

**Table 1:** Approved therapies and associated biomarkers in colorectal carcinoma.

Therapy	Trade Name	FDA Approval Year and References	Cellular Target	Indication/Biomarker
Pembrolizumab	Keytruda	2017 [92]	PD-1	High MSI/MMR deficient
Nivolumab + Ipilimumab	Opdivo + Yervoy	2018 [27]	PD-1, CTLA4	High MSI/MMR deficient
Bevacizumab	Avastin, Altuzan	2004 [54]	VEGF	All mCRC, in combo w/chemo
Aflibercept	Zaltrap, Eylea	2012 [62]	VEGF, PGF	All mCRC, in combo w/chemo
Ramucirumab	Cyramza	2015 [47]	VEGF, HER2/neu	All mCRC, in combo w/chemo
Regorafenib	Stivarga	2012 [64]	Multi RTK	Salvage therapy
Cetuximab	Erbitux	2004 [78,93]	EGFR	*KRAS, NRAS, BRAF* WT, in combo w/chemo
Panitumumab	Vectibix	2006 [94]	EGFR	*KRAS, NRAS, BRAF* WT, in combo w/chemo

PD-1—programmable death protein 1; CTLA-4— cytotoxic T-lymphocyte-associated protein 4; VEGF—vascular endothelial growth factor; PGF—placental growth factor; HER2—human epidermal growth factor 2; RTK—receptor tyrosine kinase; EGFR—epidermal growth factor receptor; MSI—microsatellite instability; MMR—mismatch repair; mCRC—metastatic colorectal cancer, WT—wild type.
